# Alcohol use in early and late adolescence among the Birth to Twenty cohort in Soweto, South Africa

**DOI:** 10.3402/gha.v6i0.19274

**Published:** 2013-01-24

**Authors:** Leane Ramsoomar, Neo K. Morojele, Shane A. Norris

**Affiliations:** 1 School of Public Health, Faculty of Health Sciences, University of the Witwatersrand, Johannesburg, South Africa; 2 Alcohol and Drug Abuse Research Unit, Medical Research Council (MRC), Cape Town, South Africa; 3 Developmental Pathways for Health Research Unit, School of Clinical Medicine, Faculty of Health Sciences, University of the Witwatersrand, Johannesburg, South Africa

**Keywords:** adolescent alcohol use, alcohol prevalence, maternal correlates, socio-economic status

## Abstract

**Background:**

Alcohol is a risk factor for the leading causes of mortality and morbidity among young people globally. Youth drinking, initiated in early adolescence and continued into early adulthood, is influenced by maternal socio-demographic factors and maternal education. Limited prospective data exists in South Africa on the prevalence of alcohol use during adolescence and adolescent and maternal socio-demographic correlates.

**Objective:**

To examine the prevalence of lifetime alcohol use during early (13 years) and late (18 years) adolescence in Soweto, South Africa, and its association with child and maternal socio-demographic factors.

**Methods:**

Data on alcohol use in early adolescence (age 13 years) and late adolescence (age 18 years) were collected using self-completed pen and paper and self-completed computer-based questionnaires, respectively. Univariate analyses were conducted on child (gender and number of school years repeated by grade 7), maternal socio-demographic correlates (education, marital status, and age), and household socioeconomic status (SES). Bivariate logistic regression analyses examined associations between alcohol use and all child and maternal socio-demographic factors. Multivariate logistic regression analyses were conducted on all the variables found to be significantly (*p*<0.10) associated with alcohol use to examine the predictive value on alcohol use at early and late adolescence.

**Results:**

Lifetime alcohol use increased from 22% at early adolescence to 66% at late adolescence. In multivariate analyses, gender, maternal education, and SES predicted lifetime alcohol use at early adolescence, while gender, maternal education, marital status, and SES were predictive of the same at late adolescence.

**Conclusion:**

This study aids researchers and practitioners to identify maternal and child socio-demographic risk profiles for alcohol use to inform policies and programmes.

Alcohol is a risk factor for leading causes of mortality and morbidity among young people. Globally, the harmful use of alcohol accounts for 2.5 million deaths (4% of total), and 69.4 million (4.5% of total) disability-adjusted life years (DALYs) (1, 2). In the 15–29 age group, 9% of total deaths are alcohol-related annually.

Safe levels of consumption have not been established for adolescent populations. This is unsurprising, given that adolescents are physiologically and psychologically more vulnerable to the effects of alcohol. Physiologically, their smaller body sizes result in a lower threshold for the effects of alcohol. Behaviourally, the disinhibitory effect of alcohol makes adolescents vulnerable to a range of risk behaviours including sexual risk, interpersonal violence, traffic-related accidents, unintentional injuries, and death (3, 4). Given that safe levels of alcohol use have not been established in adolescent populations, any use of alcohol, particularly in early adolescence, may be a predictor of later alcohol problems (5). Evidence from the South African National Youth Risk Behaviour Surveys conducted in 2002 and 2008 indicate that 49.1% of learners had drunk at least one or more drinks of alcohol in their lifetime (6). In 2008, this increased slightly to 49.6% (7). Of note is that in both surveys, 12% of learners reported having their first drink before 13 years of age (6, 7). This is of particular concern given that early initiation of alcohol is associated with substance abuse problems later in life (5).

Further to early initiation of alcohol use, the same surveys indicate that harmful/hazardous alcohol use (defined as ≥5 standard alcoholic drinks per day for males and ≥3 drinks per day for females) is also a significant problem for South African youth (6, 7). Nationally, males report more binge drinking than females overall (4, 6, 7). However, binge drinking among females has increased significantly (4, 6, 7). This early initiation of alcohol use, coupled with the harmful/hazardous use of alcohol found among South African youth, has serious consequences for public health. Hence, identifying correlates of alcohol use particularly during early adolescence is vital for prevention and intervention programmes.

Internationally there is increased focus on adolescent alcohol use for several reasons. First, adolescence constitutes a tenuous period when young people face several developmental and social challenges which place them at risk, for example, physical violence, traffic accidents, unintentional injuries, and death (4, 8). Second, alcohol use has been associated with other health risk behaviours such as smoking, other drug use, and sexual risk behaviours (9–11). Third, alcohol use initiated during adolescence can extend into later life and result in substance use disorders (5, 6).

Previous studies have shown that factors including the individual, family, and SES influence adolescent behaviour (12, 13). Specifically, the role of the mother has been examined in relation to several adolescent behaviours, including sexual risk, drug use, psychopathology, and alcohol use (12, 14, 15).

In South Africa, where adolescent drinking and early initiation of alcohol use is showing an increasing trend (4), limited research exists on adolescent and maternal socio-demographic variables as correlates for alcohol use. Of the limited studies conducted, researchers argue that it may be more pragmatic to target adolescent personal attributes, and peer and parental level factors than the social environment for prevention planning (11). On the contrary, social factors, such as living in communities with limited alcohol policing, easy access to alcohol, and low religiosity (16, 17), have also been cited as targets for prevention planning.

Flisher et al. (17) examined substance use among high school students in Cape Town, South Africa, and found lower rates of substance use, including alcohol use, among *black*^1^ females. They advocate for the importance of recognising demographic factors, such as race and gender as correlates of alcohol use in tailoring alcohol prevention programmes.

Global evidence indicates that males outnumber females with regard to frequency of alcohol use, binge drinking, and alcohol use disorders (2). This is consistent with evidence from the South African studies (5–7).

Regarding the role of SES on adolescent alcohol use, a review of 28 studies internationally found no clear pattern of associations between SES and alcohol consumption in adolescence. Five found positive associations, that is, high SES was related to high alcohol use; five reported negative associations, that is, low SES was related to higher alcohol use. Sixteen studies found no association between SES and alcohol use (18). While much evidence has indicated that adolescents with low SES have a higher inclination for alcohol use (19), other research indicates that adolescents from higher SES categories may also be at risk for substance use (including alcohol use) disorders (20). However, other literature shows differences by developmental stage in the association between SES and substance use. Specifically, adolescents with low SES were more likely than adolescents with higher SES to engage in substance use, while for adults the opposite was true (21).

Previous research has found that parental marital status is a key influencing factor in adolescent alcohol use, that is, adolescents who come from families where parents were separated or had divorced had a higher inclination for alcohol use (22). Similarly, evidence regarding the influence of maternal educational status has consistently revealed that mothers with higher educational levels are less likely to have adolescent children who use alcohol (16, 23).

All these studies provide a window into the role of socio-demographic factors in adolescent alcohol use, but they also have important limitations. Many are school-based studies that do not include youths outside of the school system who may face compounding risk factors for substance use. Flisher and Chalton (24) found that in-school youths were less likely to use substances and engage in sexual risk behaviours (the latter being girls only) than adolescents who dropped out of school. Moreover, in the South African context, many studies on adolescents capture alcohol use at only one point in time and, to the knowledge of the authors, none have examined the role of maternal socio-demographic factors in association with alcohol use.

The present study seeks to address some of these limitations by examining alcohol use in a community sample, comprising both in-school and out-of-school adolescents and the association of maternal correlates and alcohol use at two key developmental stages (early and late adolescence). Examining alcohol use at two time points enables one to demonstrate the enduring effect of maternal and child socio-demographic correlates on alcohol use behaviour. Knowledge of demographic correlates assists researchers and practitioners in identifying sub-groups of adolescents with specific maternal (e.g. low education, being a single mother) and child (e.g. gender, repetition of school grades) socio-demographic risk profiles.

## Hypotheses

This study tested three hypotheses:

1. Lower maternal education is associated with having children with a higher inclination for alcohol use during adolescence.
2. Lower SES is associated with having children with a higher inclination for alcohol use during adolescence.
3. Children of women who are not married (single not living together) at birth have a higher inclination for alcohol use during adolescence than children whose mothers are married at birth.

## Methods

### Study population

The study sample comprised of singleton children and their mothers from a birth cohort study, the Birth to Twenty (Bt20) study. This birth cohort study follows 3,273 children and their families in Soweto, Greater Johannesburg, in the Gauteng Province of South Africa. The study enrolled mothers who were 6 months pregnant with their children at the study inception. As the children were born 7 weeks after Nelson Mandela's release from prison in February 1990, they became colloquially known as ‘Mandela's children’ (25). The township of Soweto is the most populous black urban residential area in the country, with approximately 1 million people. Having originated in 1903, Soweto was the site of the 1976 uprising when school children protested against the *apartheid* 2 system in South Africa. The Bt20 study aims to track child and adolescent health and development from birth to early adulthood, along several domains – physical, social, and psychological. The retention rate of the overall cohort is 70%, with the highest attrition rate occurring during the infant years, due to permanent out-migration of mothers to rural areas following the delivery of their babies (25). Black children comprised the major race group in the study sample (78.5%), followed by coloured (11.7%), white (6.7%), and Indian (3.5%) children. The mean age of the biological mothers of the index children was 25.9 years old, and the ages ranged from 13 to 48 years old. Ethical clearance for the study was obtained under the Bt20 study from the University of the Witwatersrand Committee for Research on Human Subjects, protocol no. M080320.

### Alcohol use and socio-demographic assessment

Table 1 presents operational definitions of the variables measured in this study.

**Table 1:** Operational definitions of variables used at Wave 1 (year 13) and Wave 2 (year 18) of the analytical sample

Variable name	Variable source	Variable operationalisation	Variable coding
Child gender	Baseline Bt20 Demographic Questionnaire	Gender of child	Male=0 Female=1
School years repeated by grade 7	Wave 1 and 2 (year 13 and 18) Adolescent Questionnaire	Total number of ‘repeat’ school years up to grade 7	No school years repeated=0 1 school year repeated=1 2 school years repeated=2
Lifetime alcohol use	Wave 1 and 2 (year 13 and 18) Adolescent Questionnaire	Ever drunk alcohol in lifetime	No=0 Yes=1
Household SES	Baseline Bt20 Demographic Questionnaire	Asset index based on a list of eight assets in the baseline household. Scores for all variables were added to obtain a value from 0 to 7, and then recoded into five SES categories	Lowest=0 (0, 1, 2 assets) Low=1 (3 assets) Medium=2 (4 assets) Higher=3 (5 assets) Highest=4 (6, 7, 8 assets)
Maternal age	Baseline Bt20 Demographic Questionnaire	Continuous data recoded into age categories that are reflective of maternal age range within the sample	13–19 years=1 20–24 years=2 25–29 years=3 30–34 years=4 35–39 years=5 40–49 years=6
Maternal education	Baseline Bt20 Demographic Questionnaire	Original six categories of maternal education included	No schooling/less than grade 5 education=1 Primary=2 Secondary=3 Post-school (i.e. diploma less than 1 year; diploma 2–3 years; 3–4 year degree; masters degree; PhD; university not specified)=4
Maternal marital status	Baseline Bt20 Demographic Questionnaire	The original variable was recoded into a binary variable based on the frequency of distribution of maternal marital status in the sample	single or not living together=0 married (any definition) or living together;=1

Demographic information on the mothers and children were collected at/or within the 3 years following the birth of the child. At early adolescence (Wave 1 of the present study) risk-behaviour (such as tobacco and lifetime alcohol use) data were collected using a self-administered paper-based questionnaire. At late adolescence (Wave 2 of the present study), other risk behaviours such as tobacco, sexual activity, and detailed alcohol use (frequency and patterns of drinking, alcohol use disorders, and peer/best friend drinking) data were collected using a self-complete computer-based questionnaire. The interviewer-administered questionnaires included a wide range of indicators inclusive of socio-demographic factors, community norms, household and family circumstances, education, parent–child and peer relationships, and parental monitoring (25).

### Statistical analysis

The analytical study sample consists of two waves of cross-sectional data from the birth cohort study, which mark two developmental periods and are contextualised within this study as early adolescence (13 years) and late adolescence (18 years). Data collected from all participants on alcohol use at the early and late adolescence time points were analysed using the *Statistical Package for the Social Sciences 20* (IBM SPSS Statistics; version 20; New York, USA). Univariate frequency analyses were conducted on the demographic variables including child gender, the number of schooling years repeated by grade 7, household SES, and mother's years of education, age, and marital status at or within the 3 years following the birth of the child. Household SES was calculated based on an asset index derived from a listing of household assets (home type, home ownership, electricity in home, television, car, fridge, washing machine, phone). The use of an asset indicator as a proxy measurement for SES has been validated in developing country contexts (26–28). Asset scores were generated through an additive index, by attributing a score of 1 to assets which people owned and a score of 0 to assets which participants did not own. Participant's responses were scored based on their asset scores and ranked as ranging from 1 (lowest) to 5 (highest) SES categories. Asset scores of 0, 1, and 2 fell into the lowest SES category, 3 fell into the low SES category, 4 into medium SES category, 5 into high SES category, and 6 or 7 into the highest SES category. Bivariate logistic regression analyses were conducted to assess the associations between SES, child and maternal socio-demographic variables, and lifetime alcohol use (measured by ever having had a drink in their lifetime) at both early and late adolescence. Finally, multivariate logistic regression analyses were conducted on the variables found to be significantly (p<0.10) associated with alcohol use in the bivariate logistic regression analyses in order to examine the predictive value of these socio-demographic variables on alcohol use at early and late adolescence.

## Results

Total sample sizes for participants at early and late adolescence on whom socio-demographic and alcohol use data were collected were 1621 and 1735, respectively. Socio-demographic characteristics of the child participants at early and late adolescence are presented in Table 2. Females comprised just over half the study sample at early (52%) and late (54%) adolescence. The majority of the participants at both early (74%) and late (75%) adolescence had not repeated any schooling years by grade 7. Twenty-two percent of the sample at early adolescence and 66% at late adolescence had ever used alcohol in their lifetime. Regarding household SES, 16 and 15% of the sample at early and late adolescence, respectively, fell within the lowest wealth category (poorest). A total of 13 and 14% of the sample at early and late adolescence, respectively, fell within the highest wealth category (wealthiest). The largest group (33 and 34% at early and late adolescence, respectively) fell into the medium SES category.

**Table 2:** Socio-demographic characteristics of the analytical sample at year 13 and year 18

	Analytical sample			

	Early adolescence (*n*=1689)		Late adolescence (*n*=1735)	
	
	*n*	%	*n*	%
Child gender				
Male	762	48	758	46
Female	839	52	893	54
School years repeated by grade 7^a^				
0	1116	74	1162	75
1	331	22	337	22
2	55	4	59	4
Lifetime alcohol use	373	22	1140	66
Household SES (wealth category)				
Lowest	230	16	229	15
Low	246	17	257	17
Medium	493	34	496	33
High	304	21	310	21
Highest	194	13	203	14

a Totals do not add up to 100% due to rounding.

The characteristics of the mothers are presented in Table 3. At the time when the children were enrolled into the study, the largest proportion of mothers (53%) was between 20 and 29 years. Seventy-nine percent of mothers had secondary school education, and 62% were unmarried (single or not living together).

**Table 3:** Maternal socio-demographic characteristics at the time of the birth of the Bt20 participant

	*n*	%
Age		
13–19 years	278	18
20–24 years	468	29
25–29 years	386	24
30–34 years	285	18
35–39 years	143	9
40–49 years	34	2
Education		
No schooling/less than grade 5	81	6
Primary	93	6
Secondary	1169	79
Post-school	133	9
Marital status		
Not married	987	62
Married	596	38

Table 4 shows the results of the bivariate logistic regression analyses. In early adolescence, males were more likely than females (OR=1.507; 95% CI=1.187–1.914) to have ever drunk alcohol in their lifetime. The same is true of late adolescence (OR=1.397; 95% CI=1.139–1.714). During early adolescence, those who had repeated 2 years of school by grade 7 were significantly more likely that those who had not repeated any school years by grade 7 to have used alcohol (OR=2.518; 95% CI=1.039–6.001). There were no significant associations between the number of school years repeated by grade 7 and alcohol use at late adolescence.

**Table 4:** Bivariate logistic regression analyses of lifetime alcohol use, SES, and child and maternal socio-demographic characteristics

	Early adolescence			Late adolescence		

Lifetime use of alcohol	OR	95% CI	*p*	OR	95% CI	*p*
Child gender	1			1		
Male	1.507	1.187–1.914	0.001	1.397	1.139–1.714	0.001
No. of school years repeated by grade 7						
0	1			1		
1	2.249	0.952–5.314	0.065	1.451	0.856–2.459	0.167
2	2.518	1.039–6.100	0.041	1.443	0.824–2.528	0.199
Maternal age at birth of the child						
13–19	1			1		
20–24	3.544	1.051–11.94	0.041	1.222	0.588–2.539	0.591
25–29	3.063	0.918–10.21	0.069	0.958	0.471–1.948	0.906
30–34	3.007	0.898–10.07	0.074	1.115	0.546–2.276	0.765
35–39	2.361	0.695–8.012	0.168	1.103	0.532–2.288	0.793
40–49	2.316	0.658–8.158	0.191	1.158	0.539–2.490	0.707
Maternal education at birth of the child						
No schooling/less than grade 5	1			1		
Primary	0.237	0.105–0.537	0.001	0.308	0.103–0.921	0.035
Secondary	0.594	0.321–1.098	0.097	0.369	0.220–0.617	0.000
Post-school	0.608	0.411–0.898	0.012	0.473	0.307–0.730	0.001
Maternal marital status at birth of the child						
Not married	1			1		
Married	1.047	0.818–1.340	0.716	0.726	0.587–0.897	0.003
Household SES						
Lowest	1			1		
Low	0.601	0.384–0.940	0.026	0.793	0.432–0.975	0.037
Medium	0.553	0.354–0.863	0.009	0.853	0.455–1.008	0.055
High	0.682	0.469–0.993	0.046	0.652	0.382–0.777	0.001
Highest	0.693	0.460–1.044	0.079	1.131	0.665–1.459	0.940

Regarding maternal age, mothers between 20 and 29 years (OR=3.544; 95% CI=1.051–11.94) were significantly more likely than younger mothers (13–19 years old) to have had an adolescent child use alcohol by age 13. However, there were no significant associations between maternal age and alcohol use at late adolescence. Mothers with primary (OR=0.237; 95% CI=0.105–0.537) and post-school education (OR=0.608; 95% CI=0.411–0.898) were significantly less likely than mothers with no/less than grade 5 education to have had a child use alcohol in early adolescence. In late adolescence, mothers with primary (OR=0.308; 95% CI=0.103–921), secondary (OR=0.369; 95% CI=0.220–0.617), and post-school (OR=0.473; 95% CI=0.307–0.730) education were significantly more likely than mothers with no/less than grade 5 education to have had an adolescent child use alcohol. Marital status was not significantly associated with alcohol use in early adolescence, while at late adolescence, married mothers were significantly less likely (OR=0.726; 95% CI=0.587–0.897) than non-married mothers to have a child use alcohol.

Household SES was significantly associated with alcohol use at early and late adolescence. Specifically, at early adolescence those participants from low (OR=0.601; 95% CI=0.384–0.940), medium (OR=0.553; 95% CI=0.354–0.863), and high (OR=0.682; 95% CI=0.469–0.993) SES categories were significantly less likely than those adolescents from the lowest SES households to have ever drunk alcohol in their lifetime. The same was true for late adolescence (see Table 4).

Table 5 shows the results of multivariate logistic regression analyses. All variables found to be significantly (p<0.10) associated with alcohol use in the bivariate logistic regression analyses were included in the multivariate logistic regression. Gender was predictive of alcohol use. Males were more likely to have ever drunk alcohol in their lifetime at both early (OR=1.372; 95% CI=1.054–1.7861) and late adolescence (OR=1.387; 95% CI=1.103–1.745) than females. There were no significant associations between maternal age and alcohol use at early or late adolescence.

**Table 5:** Multivariate regression analyses of lifetime alcohol use and child and maternal socio-demographic characteristics

	Early adolescence			Late adolescence		

Lifetime use of alcohol	OR	95% CI	*p*	OR	95% CI	*p*
Child gender	1			1		
Male	1.372	1.054–1.786	0.019	1.387	1.103–1.745	0.005
No. of school years repeated by grade 7						
0	1			1		
1	2.261	0.872–5.861	0.093	1.335	0.737–2.418	0.341
2	2.489	0.872–5.861	0.067	0.489	0.737–2.418	0.341
Maternal age at birth of the child						
13–19	1			1		
20–24	3.966	0.872–18.026	0.075	1.040	0.428–2.526	0.931
25–29	3.623	0.811–16.178	0.092	0.682	0.289–1.608	0.382
30–34	3.225	0.726–14.323	0.124	0.778	0.332–1.821	0.563
35–39	2.436	0.543–10.927	0.245	0.741	0.313–1.759	0.497
40–49	2.211	0.475–10.306	0.312	0.745	0.304–1.825	0.519
Maternal education at birth of the child						
No schooling/less than grade 5	1			1		
Primary	0.312	0.127–0.768	0.011	0.381	0.106–1.368	0.139
Secondary	0.771	0.394–1.510	0.449	0.360	0.204–0.633	0.001
Post-school	0.596	0.387–0.920	0.019	0.488	0.306–0.778	0.003
Maternal marital status at birth of the child						
Not married	1			1		
Married	0.984	0.798–1.349	0.922	0.684	0.498–0.844	0.001
Household SES						
Lowest	1			1		
Low	0.681	0.417–1.112	0.125	0.837	0.535–1.309	0.435
Medium	0.596	0.366–.970	0.037	0.897	0.579–1.389	0.627
Higher	0.672	0.445–1.016	0.059	0.653	0.443–0.963	0.032
Highest	0.728	0.467–1.135	0.161	1.23	0.803–1.893	0.339

Regarding maternal education, at early adolescence, children with mothers who had had at least a primary school education (i.e. completed grade 5) were significantly less likely (OR=0.312; 95% CI=0.127–0.768) to have ever drunk alcohol, compared to children with mothers with no or less than grade 5 education. In addition, motherswho had post-school education [i.e. diploma (less than 1 year), diploma (2–3 years), 3–4 year degree, master's degree, or a PhD] were significantly less likely (OR=0.596; 95% CI=0.387–0.920) to have had children who had ever drunk alcohol in early adolescence than those with mothers with less than grade 5 or no education.

At late adolescence, maternal education was also predictive of adolescent alcohol use. Specifically, children with mothers who had secondary (OR=0.360; 95% CI=0.204–0.633) and post-school (OR=0.488; 95% CI=0.306–0.778) education were significantly less likely than children with mothers who had no schooling/less than grade 5 to have ever drunk alcohol. Significant associations emerged between maternal marital status and alcohol use in late adolescence only, with children with married mothers less likely (OR=0.684; 95% CI=0.498–0.844) to have ever used alcohol, compared to children with non-married mothers.

Finally, household SES was predictive of lifetime alcohol use. At early adolescence, participants from medium (OR=0.596; 95% CI=0.366–0.970) and higher (OR=0.672; 95% CI=0.445–1.016) SES categories were less likely than participants from the lowest SES category to have ever drunk alcohol in their lifetime. The same is true for late adolescence where participants from the higher SES category (OR=0.653; 95% CI=0.443–0.963) were less likely than the participants from the lowest SES adolescents to ever have drunk alcohol in their lifetime.

## Discussion

This paper examined lifetime alcohol use among a birth cohort in Soweto, South Africa, in early (13 years) and late (18 years) adolescence and its association with household SES as well as child and maternal socio-demographic factors. Specifically, we examined child gender, the number of years the adolescents repeated schooling by grade 7, maternal age, education, marital status, and household SES in association with lifetime alcohol use at these two developmental stages.

Consistent with national and international literature (4, 6, 7), this study found gender differences in rates of alcohol use at both early and late adolescence, indicated by the higher prevalence of adolescent males who drank alcohol in their lifetime and significant associations between gender and alcohol use revealed by bivariate and multivariate analyses, respectively.

The significant association found in early adolescence between the repetition of 2 years of schooling by Grade 7 and lifetime alcohol can be explained by previous research which indicates that poor educational attainment has been associated with substance use (24).

The marginally significant associations found in the multivariate analysis between the repetition of 2 years of schooling by Grade 7 and lifetime alcohol can potentially be confounded by the effect of SES and maternal education (28).

This study also contributes to a body of literature on the relationship between SES and alcohol use. Present findings are consistent with evidence and confirm the study hypotheses that SES is predictive of adolescent alcohol use. Both bivariate and multivariate results corroborate much existing evidence that low SES is related to higher alcohol use (18, 29). In particular, at early adolescence, participants from medium and higher SES categories were less likely to have ever used alcohol in their lifetime than adolescents from the lowest SES category. The latter finding is also true for late adolescence. Similarly, maternal education had consistent predictive value in adolescent alcohol use at early and late adolescence; the higher the maternal education, the less likely adolescents were to have ever drunk alcohol in their lifetime. Potential explanations for these findings are that adolescents from lower SES categories and/or whose mother's education level is lower may be less likely than higher SES categories, with higher maternal education, to be educated about alcohol-related risks and harm (24). They may also have more access to unregulated sale of alcohol than higher SES adolescents (9) and live in areas where alcohol outlet density is higher (30).

The significant associations found between maternal education and alcohol use highlight the protective role of maternal education. At early adolescence, both maternal primary and post-school education were significantly associated with and predictive of alcohol use. At late adolescence, the same was true of the association between mothers with secondary and post-school education and their adolescent children's alcohol use. These findings could be explained by other research, which suggest that more educated mothers not only engage in healthier behaviour but also have more disposable income to afford more or better health protection (quality food, health care, live in safer neighbourhoods) for their children (31) than mothers who may be less educated with less disposable income. Mothers with higher educational attainment may also, by virtue of their own health education, be better positioned to provide health promotion and education and model more health behaviours than mothers who are less educated (31). Consequently, educating mothers to a higher level may also have benefits for preventing alcohol use by their adolescent children.

Finally, the significant associations between maternal marital status and alcohol use in late adolescence only, partially confirms the study hypothesis that adolescents of unmarried mothers have a higher inclination for alcohol use than adolescents of married mothers. The particular association between maternal marital status and alcohol use among *older* adolescent children may be explained by research which indicates that the lack of a biological father can have negative implications for the socialisation of children (32). Given that more discipline and adolescent social supervision may be required in later adolescence than in earlier adolescence, and single mothers have been found to exert less authority and provide less discipline to their children than married parents (33), the absence of a biological father may have more profound negative effects on children's alcohol use behaviour later, rather than earlier in life.

Taken together, these findings have important implications for planning and programmes. Specifically, programmes targeting risk and protective factors for adolescent alcohol use must take account of the role of gender, SES, and maternal education in adolescent alcohol use. The findings also point to the need for mothers (with low education), boys, and children from lower SES to be targeted (albeit differentially at different stages) as intervention points for adolescent alcohol prevention initiatives. Moreover, future research is required to examine potentially relevant socio-demographic factors in tailoring adolescent alcohol prevention programmes. Finally, maternal education and SES may only partially account for the association between socio-demographic correlates and adolescent alcohol use. The absence of the biological father, coupled with the influence of other determinants (peers, community contexts), may further explain adolescent alcohol use.

There are limitations to this study notably our inability to consider the role of the father in adolescent alcohol use. Low father involvement was due, in part, to the migrant labour system in apartheid South Africa, which disrupted the structure of black families (34). Bearing children outside a marital arrangement was relatively normative in these contexts, resulting in children being born with very low father involvement in the Birth to Twenty cohort (25). This explains why the majority of mothers were single parents and, therefore, the primary contact for the study. Future research from the birth cohort is required to understand the presence and potential influence of a father figure on adolescent risk behaviours. Furthermore, as with any birth cohort study, loss to follow up is a limitation. Another limitation is the definition of the outcome measure (ever drunk) as a selfreported outcome measure. This is subject to socially desirable responses which potentially result in an over-/underestimation of alcohol use. The recognition of marital status in South Africa under many arrangements, including civil unions and customary unions, and co-habitation makes the standard definition of marriage used in this study a potential limitation.

Additionally, we acknowledge the potential changes that may have occurred in maternal marital status and education from study inception to the survey waves. However, future longitudinal analyses are required to examine the effect of changing maternal socio-demographic characteristics on adolescent alcohol behaviour, as the aim of this paper was to examine the role of child and maternal socio-demographic correlates at birth in adolescent alcohol use. Finally, we recognize that lifetime use of alcohol as the only outcome measure is a limitation. Nevertheless, given that alcohol use is initiated in adolescence, this may be an important marker of future alcohol use. Future studies employing a life course approach to the development of adolescent alcohol behaviours are envisaged to examine the precision of lifetime alcohol use as a measure of later alcohol problems in this birth cohort.

## Conclusion

This study makes a contribution to informing tailored prevention programmes for adolescent alcohol use at important stages in their developmental process. Future research is required to understand the interactions between psychosocial (social support, parenting styles, monitoring) and socio-demographic (age, SES) factors that may play a role in predicting adolescent alcohol use.

## Footnotes

1 The terms ‘white, black, Indian/Asian, and coloured’ originate from the *apartheid* era. They refer to demographic markers and do not signify inherent characteristics. Their continued use in South Africa is retained to track transformation and to identify vulnerable sections of the population to be targeted for prevention and intervention programmes.

2 The *apartheid* era was characterised by a separatist regime, during which the government of the day legitimised the differentiation of people based on race difference, whites, Indians, coloured (mixed ancestry), and blacks (of African descent).
